# Childhood maltreatment, psychopathology, and the development of hippocampal subregions during adolescence

**DOI:** 10.1002/brb3.607

**Published:** 2016-11-30

**Authors:** Sarah Whittle, Julian G. Simmons, Sylke Hendriksma, Nandita Vijayakumar, Michelle L. Byrne, Meg Dennison, Nicholas B. Allen

**Affiliations:** ^1^ Melbourne Neuropsychiatry CentreDepartment of PsychiatryThe University of Melbourne and Melbourne HealthParkvilleVic.Australia; ^2^ Melbourne School of Psychological SciencesThe University of MelbourneParkvilleVic.Australia; ^3^ Department of PsychologyUniversity of OregonEugeneORUSA; ^4^ Department of PsychologyUniversity of WashingtonSeattleWAUSA

**Keywords:** brain development, child abuse, longitudinal, mental health, neuroimaging

## Abstract

**Introduction:**

It is well established that childhood maltreatment has a detrimental impact on the brain, particularly the hippocampus. However, the hippocampus is a functionally and structurally heterogeneous region, and little is known about how maltreatment might affect hippocampal subregion development throughout important periods of plasticity. This study investigated whether childhood maltreatment was associated with the development of hippocampal subregion volumes from early to late adolescence. It also investigated associations between onset of psychiatric disorder and hippocampal subregion volume development.

**Methods:**

One hundred and sixty‐six (85 male) adolescents took part in three magnetic resonance imaging assessments during adolescence (mean age at each assessment: 12.79 [*SD* 0.43] years, 16.70 [*SD* 0.52] years, and 19.08 [*SD* 0.46] years), provided a self‐report of childhood maltreatment, and were assessed for Axis I psychopathology.

**Results:**

Childhood maltreatment was associated with the development of right total and left cornu ammonis 4 (CA4‐DG) volumes from early to late adolescence. Early and late onset psychopathology was associated with the development of right presubiculum and right cornu ammonis 1 (CA1) volumes, respectively. Maltreatment findings appeared to be specific to males, whereas psychopathology findings appeared to be specific to females.

**Conclusions:**

These findings provide evidence for possible deleterious effects of childhood maltreatment and early onset psychiatric disorder on the development of different subregions of the hippocampus. Altered development of the right CA1, on the other hand, might precede the development of late‐adolescent onset psychopathology. Our results highlight the importance of considering development in research examining associations between stress, mental illness, and hippocampal morphology.

## 1 Introduction

Child maltreatment has negative consequences for those who are exposed and society more broadly (Gilbert et al., 2009). Extant literature has demonstrated that experiencing childhood maltreatment increases the risk of developing psychopathology in childhood and later in life (De Bellis, 2001; Teicher et al., 2003). Given evidence that childhood maltreatment changes the functioning of neurobiological stress systems (Dedovic, Duchesne, Andrews, Engert, & Pruessner, 2009; Teicher et al., 2003), altered neurobiological stress reactivity could be one of the mechanisms by which childhood maltreatment contributes to the development of psychopathology.

The hippocampus plays a key role in neurobiological stress systems. With a protracted ontogeny, relatively high levels of glucocorticoid receptors and persistent postnatal neurogenesis, the hippocampus is particularly vulnerable to the effects of stress (Teicher et al., 2003). Furthermore, there is some suggestion that stress‐related effects on hippocampal morphology may render individuals vulnerable to the development of psychopathology (Vythilingam et al., 2002), highlighting the importance of understanding the effects of stress on hippocampal structure.

In adults with a history of childhood maltreatment, multiple studies have found decreased volume of the hippocampus, particularly in the left hemisphere (McCrory, De Brito, & Viding, 2010; Teicher, Anderson, & Polcari, 2012). Results have been less consistent, however, for similar research in children and adolescents. Such studies have variably found no changes in hippocampal volume (De Bellis et al., 1999), increased volume (Tupler & De Bellis, 2006), or decreased volume (Edmiston et al., 2011).Two important factors may account for these discrepant findings. First, neurodevelopment in child/adolescent populations might be important to consider. We have recently provided evidence that childhood maltreatment does not necessarily reduce hippocampal volume per se, but rather has a negative effect on normative patterns of *development* (Whittle et al., 2013). Second, given that the hippocampus is a structurally and functionally heterogeneous region, it is possible that maltreatment affects the structure of some hippocampal subregions more than others.

The hippocampal formation includes the hippocampus proper (i.e., cornu ammonis [CA]), the dentate gyrus, the entorhinal cortex, and the subiculum, presubiculum, and parasubiculum (Andersen, Morris, Amaral, Bliss, & O'Keefe, 2006; Bartsch, 2012; Mayford, 2014). Based on cytoarchitecture, the hippocampus proper can be divided in four subregions: CA1, CA2, CA3, and CA4, although some consider CA4 part of the dentate gyrus. Animal studies suggest that stress primarily acts to suppress neurogenesis of dentate gyrus granule neurons, and to cause remodeling of dendrites in the CA3 (McEwen, 2002).

Only a small number of studies to date have assessed the effects of childhood maltreatment on hippocampal subregion volumes. Consistent with the animal literature, Teicher et al. (2012) found associations between childhood maltreatment and reduced volume of the left hippocampal regions CA2‐CA3 and CA4‐dentate gyrus (CA4‐DG), in addition to left subiculum and presubiculum and right CA1, in a community sample of adults. Other studies have investigated the effects of maltreatment on the volume of hippocampal subregions in adults with mental disorders. Aas et al. (2014) found that individuals with schizophrenia spectrum or bipolar disorder reporting high levels of childhood maltreatment had significantly reduced volumes of (total, left, and right combined) CA2–3 and CA4‐DG (but only in individuals with a specific gene variant). Chalavi et al. (2015) found that patients with posttraumatic stress disorder or dissociative identity disorder had significantly smaller CA1, CA2–3, CA4‐DG, subiculum, and presubiculum volumes than healthy controls, and that in the patient groups, childhood maltreatment was associated with smaller CA2–3, CA4‐DG, subiculum, and presubiculum volumes in the left hemisphere. Finally, Bøen et al. (2014) found reductions in CA1, CA2–CA3, and CA4‐DG volumes in females with borderline personality disorder in comparison to healthy control participants, however, history of maltreatment was not associated with volume of any subregion.

No research to date has investigated the effects of maltreatment on hippocampal subregion volumes in young people (i.e., children or adolescents), nor has any research investigated the effects of maltreatment on patterns of hippocampal subregion volume development using a longitudinal design. Such research is essential to understand how the effect of childhood maltreatment on specific brain regions unfolds over time during a critical period for both brain development and vulnerability to mental disorders. The aim of this study was to extend our previous work on maltreatment and hippocampal volume development in a community sample of adolescents (Whittle et al., 2013) by assessing the effects of maltreatment on hippocampal subregion volume development from early to late adolescence (extending the age range investigated in our previous work). Based on the findings outlined above, in addition to research showing that all hippocampal subregion volumes show increases during adolescence (Krogsrud et al., 2014), we hypothesized that childhood maltreatment would be associated with attenuated (or flatter growth) developmental trajectories of left hippocampal subregion volumes, in particular of CA4‐DG and the subicular regions.

Given that psychopathology has also been associated with altered hippocampal subregion volumes, and that many studies of the effects of maltreatment on hippocampal volume are performed in samples of mentally ill participants (McCrory et al., 2010), in exploratory analyses, we examined the influence of relatively early versus late onset of mental disorder in order to provide some comment on the possible direction of effects regarding psychopathology and hippocampal change. See Supporting information for a description of previous analysis of similar data from the same sample.

## 2 Materials and Methods

### 2.1 Participants

The sample described in this study was derived from a larger (*N* = 2,453) Australian cohort. Based on their scores on the Early Adolescent Temperament Questionnaire‐Revised (Capaldi & Rothbart, 1992), 415 year six primary school students were selected to be part of the study, which has previously been described in detail by Yap et al. (2011). Adolescents at the extreme ends of the temperamental distribution were oversampled to maximize interindividual differences in risk for psychopathology.

Of the selected adolescents, 245 agreed to participate in further research, and were invited to take part in brain magnetic resonance imaging (MRI) assessments at three time points, when they were aged approximately 12 (Time 1, T1), 16 (Time 2, T2), and 19 (Time 3, T3), respectively. Participants were assessed for Axis I disorders at each of these time points using the Kiddie—Schedule for Affective Disorder and Schizophrenia for School‐Aged Children: Present and Lifetime Version (Kaufman et al., 1997). Socioeconomic classification (SES) was assessed based on the Australian National University Four (ANU_4_) Scale (Jones & McMillan, 2001). A number of adolescents declined participation in the MRI assessments, resulting in 177 participants completing an MRI assessment at one or more time points. Based on visual inspection of processed MRI data, nine of these participants were excluded due to poor MRI image quality and parcellation. In addition, two participants with full‐scale IQ lower than 70 were excluded from analyses.

Following exclusions, 166 participants (*n* = 86 males) aged 11–20 years were available for analysis. Seventy‐three of these participants had three scans, 55 had two scans and 38 had one scan. The final sample also did not differ from the initial school screening sample (*N* = 2,453) on socioeconomic disadvantage (*t*_2439_ = −1.053; *p *=* *.29) or sex (Pearson's χ^2^ = 1.963; *p *=* *.743). In the sample, 76 participants met criteria for psychiatric diagnosis (see Table 1 for breakdown of disorder type). Informed consent was obtained from the child and at least one parent/guardian at each time point, consistent with the guidelines of the Human Research Ethics Committee at The University of Melbourne, Australia.

**Table 1:** Sample characteristics (mean; standard deviation)

	Male	Female	Total
T1 age (years)	12.83; 0.452	12.77; 0.394	12.79; 0.425
T2 age (years)	16.70; 0.559	16.71; 0.480	16.70; 0.518
T3 age (years)	19.10; 0.507	19.05; 0.413	19.08; 0.460
Delay time 1–2 (years)	3.80; 0.158	3.87; 0.237	3.83; 0.204
Delay time 2–3 (years)	2.40; 0.177	2.35; 0.251	2.38; 0.219
SES	58.14; 20.42	58.01; 21.36	58.08; 20.80
CTQ total score T2	33.04; 7.51	33.09; 10.67	33.07; 9.16
CTQ total score T3	31.30; 7.43	32.11; 10.07	31.70; 8.80
CTQ total score before T1	34.52; 7.99	35.05; 11.03	34.78; 9.53
Emotional abuse	8.10; 3.43	8.78; 4.70	8.43; 4.10
Emotional neglect	19.99; 4.18	20.67; 4.59	20.32; 4.08
Physical abuse	5.89; 1.62	6.09; 2.51	5.99; 2.09
Physical neglect	12.75; 1.50	12.68; 1.53	12.72; 1.51
Sexual abuse	5.00; 0.38	5.53; 2.46	5.26; 1.75
Internalizing disorder^a^ (N)	25	33	58
Externalizing disorder^b^ (N)	20	13	33
Comorbid disorder^c^ (N)	9	6	15

NB: There were no sex differences in any variable reported in this table.

T1, Time 1; T2, Time 2; T3, Time 3; SES, socioeconomic status; CTQ, Childhood Trauma Questionnaire.

a Included depressive, anxiety, adjustment, and eating disorders.

b Included behavioral (e.g., oppositional defiant disorder, conduct disorder) and substance use disorders.

c Comorbid internalizing and externalizing disorder.

### 2.2 Childhood maltreatment

Exposure to childhood maltreatment was assessed using the Childhood Trauma Questionnaire (CTQ, Bernstein & Fink, 1998) during assessments at T2, and at T3. The CTQ was developed to assess a range of traumatic experiences individuals could have been exposed to in childhood. The CTQ consists of 28 items that are rated on a 5‐point Likert scale. It measures the frequency and severity of five different types of abuse and neglect. A total maltreatment score can also be calculated. Research has shown that the CTQ is a sensitive and valid screening tool to assess childhood trauma (Bernstein, Ahluvalia, Pogge, & Handelsman, 1997; Bernstein et al., 2003). Given the highly comorbid nature of childhood maltreatment (Dong et al., 2004), the total maltreatment score was used in the analyses. Furthermore, given our aim of investigating the effects of maltreatment that occurred during childhood and prior to the first MRI assessment, we scored items only if participants indicated that they occurred or began prior to T1 (i.e., prior to calculating a total score, any item that was rated as occurring, or beginning after the age of the participant's T1 assessment was recoded as “never true” (or “very often true” in the case of reverse‐coded items). The CTQ was completed at one or both time points by 151 participants, and 144 completed the CTQ at both time points. When the CTQ was completed at both time points, data was amalgamated such that for any discrepancies in responses, the more severe rating was retained. This approach was based on evidence (1) that individuals tend to under‐report maltreatment experiences (Shaffer, Huston, & Egeland, 2008; Williams, 1994), and (2) for “recovered” memories or recall bias (Allen, 1995).

### 2.3 MRI acquisition and processing

#### 2.3.1 MRI acquisition

At each of the three time points, whole‐brain structural imaging was performed using MRI. The baseline scans were conducted in 2004 and 2005 at the Brain Research Institute in Melbourne, Australia, on a 3 Tesla GE scanner. A T1‐weighted sequence was performed with the following parameters: repetition time = 36 ms; echo time = 9 m; flip angle = 35°, field of view = 20 cm^2^, pixel matrix = 410 × 410; 124 T1‐weighted contiguous 1.5 mm‐thick slices (voxel dimensions = 0.4883 × 0.4883 × 1.5 mm). At T2 and T3, in 2010–2011 and 2012–2013, respectively, MRI‐scans were conducted at the Royal Children's Hospital Melbourne, Australia on a 3 Tesla Siemens scanner with the following parameters: repetition time = 1,900 ms; echo time = 2.24 ms; flip angle = 9°, field of view = 23 cm^2^, produced 176 T1‐weighted contiguous 0.9‐mm thick slices (voxel dimensions = 0.9 mm^3^). Although different scanners were used at T1 and T2, we have previously reported no interscanner bias for hippocampal volumes (Whittle et al., 2013, 2014). Importantly, maltreatment could not interact with scanner in any way such that any found associations between maltreatment and hippocampal subregion development could be attributed to any interscanner bias.

#### 2.3.2 Image processing

Automated measures of hippocampal subregion volumes (in addition to total hippocampal volume and intracranial volume [ICV]) were obtained using the longitudinal stream in FreeSurfer v5.3 (RRID:SCR_001847; Reuter, Schmansky, Rosas, & Fischl, 2012; Van Leemput et al., 2009).

### 2.4 Statistical analysis

Statistical analyses were conducted in SPSS version 22 (RRID:SCR_002865). Linear regression models were used to investigate the effects of childhood maltreatment (as a continuous variable), and its interaction with sex, on hippocampal subregion volumes at T1. Next, linear mixed models (LMM) were used to investigate the effect of childhood maltreatment on the development of hippocampal subregion volumes. LMM allows for the inclusion of all participant data (i.e., *n* = 166). We used full analytic models to explore the linear and quadratic effect of age, as well as interactions with sex and childhood maltreatment on volume of each subregion (tested in separate LMMs). SES and ICV were included as covariates in all analyses. A top‐down procedure was used such that higher order interaction terms were removed if nonsignificant (*p* > .05) and reduced models were rerun. For baseline (T1) and developmental analyses, the significance level was corrected using a false discovery rate (Benjamini & Yekutieli, 2001) method for each hemisphere (6 ROIs) yielding a significance level of .0204 (Narum, 2006).

We conducted follow‐up analyses to assess the association between presence of psychiatric diagnosis (any, early or late onset) and hippocampal subregion/total baseline volume and development. Early onset was defined as disorder onset at age 15 or earlier, whereas late onset was defined as disorder onset from age 16 onwards. Fifty one (*M* age onset 10.27, *SD* 3.80, 24 female) and 25 (*M* age onset 16.80, *SD* 0.82, 12 female) participants experienced an early and late onset disorder, respectively. Similar to the maltreatment analyses, we explored the linear and quadratic effect of age, as well as interactions with sex and psychiatric diagnosis on volume of each subregion (tested in separate LMMs). SES and ICV were included as covariates. The significance level was set to .0204. In addition, in order to examine whether psychiatric disorder might better explain the association between maltreatment and hippocampal subregion baseline volume or development, for models where maltreatment predicted a hippocampal subregion or total volume, we re‐ran maltreatment models including psychiatric diagnosis as a covariate. Note that all continuous variables that were used to create interaction terms were mean‐centered, and these centered variables were used in all analyses.

## 3 Results

### 3.1 Normal development

Mixed models were run to establish overall developmental trajectories in the sample. See Supporting information, for full details. In summary, results are consistent with Krogsrud et al. (2014), with nonlinear growth observed for most regions.

### 3.2 Childhood maltreatment – baseline

There was a significant interaction between sex and childhood maltreatment in the prediction of left presubiculum volume at baseline (i.e., T1: β = −.360, *t*_165_ = −2.410, *p = *.017). Separate analyses in males and females showed that maltreatment predicted larger baseline left presubiculum volumes in males (β *= *.251, *t*_80_ = 2.068, *p *=* *.043) but not females (*p *=* *.128). Maltreatment (or its interaction with sex) did not predict the baseline volume of any other region.

### 3.3 Childhood maltreatment – development

**Figure 1:**
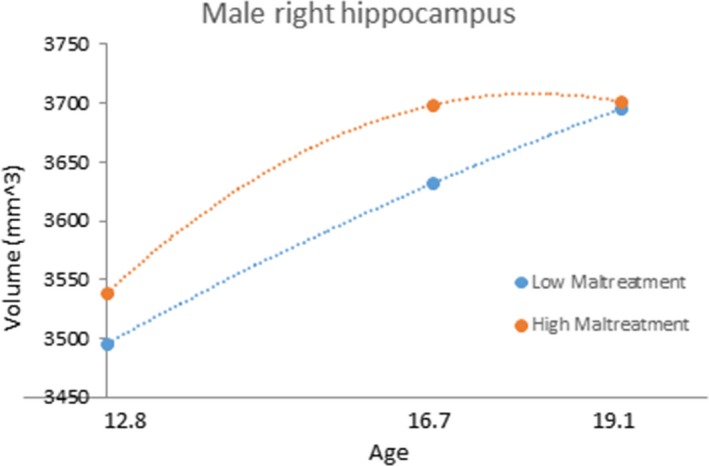
The development of total right hippocampal volume in males according to maltreatment history. The blue line represents children with low Childhood Trauma Questionnaire (CTQ) scores (−1 standard deviation; equivalent to a total score of 26.5), the orange line represents children with high CTQ‐scores (+1 standard deviation; equivalent to a total score of 48.5, equivalent to meeting for at least one form of maltreatment based on cut‐off scores provided by Walker et al. (1999)). Note that this grouping is for illustrative purposes only. Continuous CTQ total scores were used in all analyses

There was a significant interaction between the age^2^, sex, and childhood maltreatment in the prediction of right total hippocampus volume (Estimate* *= 0.548, *t*_195_ **= 2.543, *p *=* *.012). To probe the significant interaction, separate analyses were conducted for each sex; these analyses suggested that the significant interaction was driven by a stronger effect for males (Estimate* *= −0.339, *t*_95_ **= −1.823, *p *=* *.073), compared to females (Estimate = 0.178, *t*_96_ **= 1.551, *p *=* *.125). Figure 1 shows the development of the right hippocampus in males with relatively high and low levels of childhood maltreatment.

**Figure 2:**
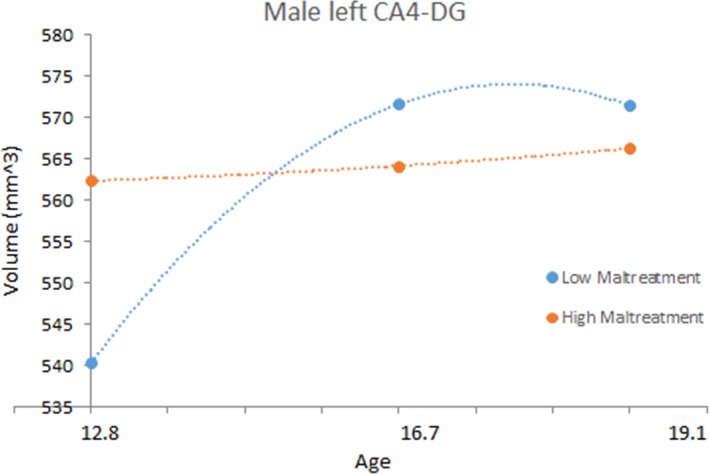
Effects of childhood maltreatment on the development of the male left CA4‐DG subregion. The blue line represents children with low Childhood Trauma Questionnaire (CTQ)‐scores (−1 standard deviation; equivalent to a total score of 26.5), the orange line represents children with high CTQ‐scores (+1 standard deviation; equivalent to a total score of 48.5, equivalent to meeting for at least one form of maltreatment based on cut‐off scores provided by Walker et al. (1999)). Note that this grouping is for illustrative purposes only. Continuous CTQ total scores were used in all analyses

There was a significant interaction between age^2^, sex, and childhood maltreatment in the prediction of the volume of the left CA4‐DG region (Estimate* *= −0.110, *t*_197_= −2.425, *p *=* *.017). Separate analyses conducted for each sex suggested that the significant interaction was driven by a stronger effect for males (Estimate* *= 0.0726, *t*_96_ **= 1.884, *p *=* *.064), compared to females (Estimate = −0.370, *t*_99_ = −1.615, *p *=* *.111). Figure 2 shows the effects of childhood maltreatment on the development of the left CA4‐DG in males. See Supporting information for female plots for the right total hippocampus and left CA4‐DG. Childhood maltreatment did not have any main or developmental effects on left total hippocampal volume, or any other hippocampal subregion volume.

### 3.4 Psychiatric diagnosis

After controlling for SES, higher levels of childhood maltreatment predicted higher rates of psychiatric diagnosis overall (*B *=* *.102, *SE* = .024, *p *<* *.001), as well as early (*B *=* *.060, *SE* = .019, *p *=* *.002) and late onset (*B *=* *.042, *SE* = .021, *p = *.042) disorders separately. These associations were not moderated by sex (*p*'s > .05).

### 3.5 Psychiatric diagnosis – baseline

Overall psychiatric diagnosis was associated with smaller volume of the right CA2–3 (β = −.215, *t*_165_ = −2.535, *p *=* *.012) and right CA4‐DG (β = −.228, *t*_165_ = −2.642, *p *=* *.009). Late onset psychiatric diagnosis was associated with smaller right subiculum volume (β = −.225, *t*_165_ = −2.605, *p *=* *.010). Overall, early and late onset psychiatric diagnosis (and their interaction with sex) was not associated with the baseline volume of any other region.

### 3.6 Psychiatric diagnosis – development

**Figure 3:**
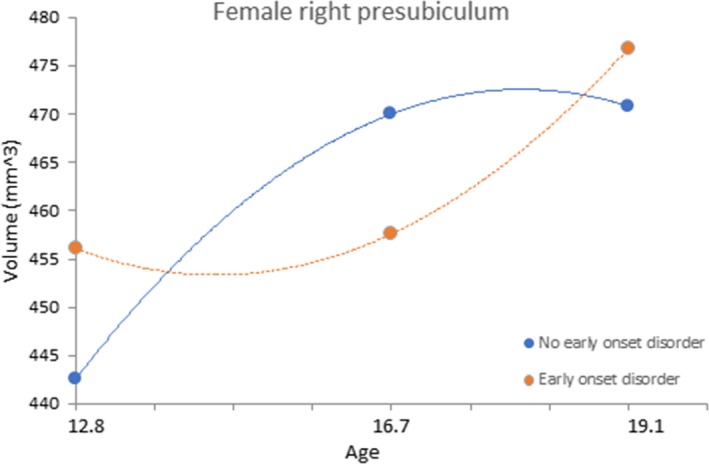
Association between early onset psychiatric disorder and the development of the female right presubiculum

**Figure 4:**
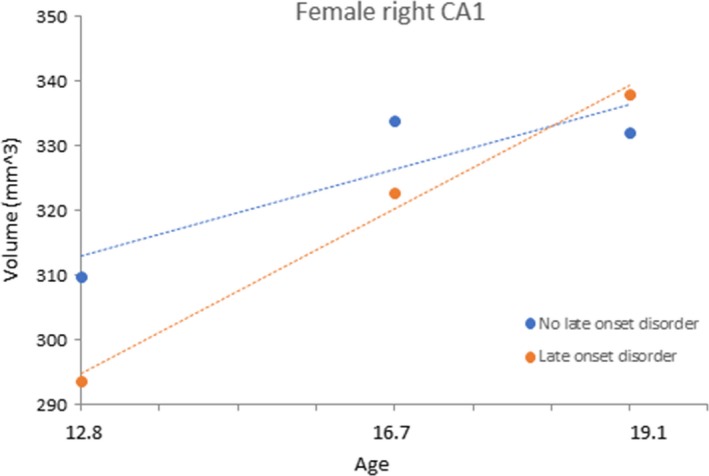
Association between late onset psychiatric disorder and the development of the female right CA1 subregion

Independent of time, overall psychiatric diagnosis was associated with smaller volume of the right CA2–3 (Estimate = 40.600, *t*_160_ = 2.582, *p *=* *.011) and right CA4‐DG (Estimate = 22.577, *t*_161_ = 2.609, *p *=* *.010) hippocampal sub‐regions. There were no effects of overall psychiatric diagnosis on trajectories of hippocampal development. When considering early and late onset psychiatric diagnoses separately, there was a significant interaction between sex, age^2^, and early onset diagnosis in the prediction of right presubiculum volume (Estimate = 2.430, *t*_207_ = 2.586, *p *=* *.011). Separate analyses conducted for each sex suggested that the significant interaction was driven by a stronger effect for females (Estimate = −1.804, *t*_101_ = −3.104, *p *=* *.003), compared to males (Estimate = 0.697, *t*_106_ = 0.924, *p *=* *.358). Figure 3 shows the association between early onset disorder and the development of the right presubiculum in females. Finally, there was a significant interaction between sex, age and late onset diagnosis in the prediction of right CA1 volume (Estimate = 4.568, *t*_199_ = 2.622, *p *=* *.010). Separate analyses conducted for each sex suggested that the significant interaction was driven by a stronger effect for females (Estimate = −3.763, *t*_99_ = −3.402, *p *=* *.001), compared to males (Estimate = 0.500, *t*_100_ = 0.369, *p *=* *.714). Figure 4 shows the association between late onset disorder and the development of the right CA1 region in females. See Supporting information for male plots for the right presubiculum and CA1 region. There were no other significant effects of psychiatric diagnosis (early or late onset) on hippocampal volumes of their growth trajectories.

Running models whereby psychiatric diagnosis and maltreatment were both included as independent variables did not change the sex by maltreatment by age^2^ effect for right hippocampal volume (*p *=* *.023), or for left CA4‐DG subregion volume (*p *=* *.016). Moreover, including maltreatment as a covariate in significant models involving psychiatric diagnosis did not change results, apart from slightly weakening the early diagnosis by sex by age^2^ effect on right presubiculum volume (*p *=* *.034).

Note that for all regions where there were significant effects of maltreatment or psychiatric diagnosis on hippocampal total or subregion development, regression analyses were performed to determine whether there were cross‐sectional effects at each time point. These analyses revealed no significant effects.

## 4 Discussion

In this longitudinal brain imaging study, we found effects of childhood maltreatment on the development of total right hippocampal and left CA4‐DG volumes. These results are consistent with our previous work with this sample showing maltreatment to affect the structure of total hippocampal volume and development (Whittle et al., 2013), and other work specifically implicating the structure of the CA4‐DG region (Aas et al., 2014; Chalavi et al., 2015; Teicher et al., 2012). Further, while the experience of a psychiatric disorder during adolescence was associated with hippocampal total and subregion volume development, these associations were different to those found for maltreatment, and analyses suggested that the experience of a psychiatric disorder did not better explain the observed maltreatment effects (and vice versa). Finally, we found that associations between hippocampal development and maltreatment were more pronounced for males, whereas associations between hippocampal development and disorder were more pronounced for females.

Childhood maltreatment was associated with larger left presubiculum volume at baseline, and growth of the left CA4‐DG in males. Regarding the latter finding, male adolescents with a history of childhood maltreatment showed a trend for retarded growth over the adolescent period. That the CA4‐DG region was implicated is consistent with animal studies, which support specific stress‐related damage to the CA4‐DG region of the hippocampus (Zach, Mrzílková, Rezacova, Stuchlík, & Vales, 2010). This region is particularly vulnerable to stress because it has a high density of multipotent stem cells and cell proliferation can be hampered by exposure to glucocorticoids (McEwen, 2002).

In general, studies have shown a greater left‐sided effect of stress on the hippocampus (Vythilingam et al., 2002). Our results for left‐sided effects for the presubiculum and CA4‐DG regions are consistent with previous maltreatment research (Chalavi et al., 2015; Teicher et al., 2012). However, childhood maltreatment was also associated with altered growth of the right total hippocampus in males. Other studies also show effects of stressors on the right hippocampus (Vermetten, Schmahl, Lindner, Loewenstein, & Bremner, 2006; Weniger, Lange, Sachsse, & Irle, 2009). Furthermore, a study in rats showed a negative effect of high doses of corticosteroids in both left and right hemisphere (Zach et al., 2010). Given the current findings, further research is required to investigate the potential significance of laterality in relation to stress and hippocampal volumes.

The experience of psychopathology was also associated with hippocampal volume development, however, the regions implicated were different to those associated with maltreatment, and analyses suggested that the experience of a psychiatric disorder did not better explain the observed maltreatment effects (and vice versa). This is interesting in light of other research suggesting that hippocampal volumetric abnormalities in psychiatric disorders (particularly depression) might be explained by the experience of maltreatment (Opel et al., 2014). However, in our data, although maltreatment was significantly associated with the development of psychopathology, our results do not provide evidence that alterations to hippocampal development mediate this association. Early onset disorder was associated with development of the right presubiculum in females. A similar pattern to the left CA4‐DG was observed, such that in early adolescence, those with an early onset disorder showed a reduced growth rate, particularly during early adolescence. It is possible that stress related to experiencing psychopathology had an impact on early adolescent presubiculum development. The presubiculum has a high density of glucocorticoid receptors (Reul & Kloet, 1985; Sarrieau et al., 1986; Stumpf, Heiss, Sar, Duncan, & Craver, 1989), making its vulnerability to stress unsurprising. Alternatively, some other stressor, or other factor (e.g., genetic) may have influenced both the development of the right presubiculum and the early onset of psychopathology. Reduced right subiculum volume and relative accelerated development of the right CA1 subregion was associated with disorder onset during late adolescence (the latter for females only). Interestingly, the difference in volume of the right CA1 subregion between females with and without late onset disorder appeared to be most marked during early adolescence (although this was not significantly significant), and reduced over time. Although speculative, these findings may indicate early hippocampal development as a vulnerability marker for disorder onset later in adolescence.

Interestingly, our findings suggest that left CA4‐DG development in maltreated males, and right presubiculum development in females with early onset disorders might “catch‐up” to “healthy” individuals by late adolescence (as suggested by a “closing in the gap” of volume differences during late adolescence – see Figures 2 and 3). Future research is needed to establish the implications of these specific patterns of hippocampal development in those individuals who have experienced childhood maltreatment and/or early onset psychiatric disorders.

The sex differences in our results are notable. Other studies investigating links between maltreatment and hippocampal subregion volumes have not tested for sex differences (Aas et al., 2014; Teicher et al., 2012) or have tested single‐sex samples (Chalavi et al., 2015). However, other studies have found support for male‐specific effects of maltreatment on hippocampal volumes (Samplin, Ikuta, Malhotra, Szeszko, & DeRosse, 2013). It may be that males are more susceptible to the neurobiological effects of childhood maltreatment than females. One possible explanation for this may be the positive effect of estradiol on hippocampal structure and development in females (De Bellis et al., 2001).

The results of this study should be seen in the light of some limitations. First, while the use of semiautomated software has many advantages, such as the ability to produce results that can be easily duplicated between research groups, there are some concerns about the reliability and validity of the method (Wisse, Biessels, & Geerlings, 2014). Future research should aim to measure hippocampal subregion volumes using alternative methods such as from high‐resolution T2‐weighted images, or from an average T1‐weighted image obtained from multiple acquisitions. Of note, of the studies published on maltreatment and hippocampal subregion volumes to date, two have relied on single T1‐weighted images for segmentation (Chalavi et al., 2015; Teicher et al., 2012) and as such, our findings can be compared with some existing work. Second, MRI scans were acquired from different MRI scanners. However, our previous work has shown no interscanner bias for the hippocampus (Whittle et al., 2014). Further, maltreatment would not interact with scanner in any way that could bias results. Third, we obtained childhood maltreatment data through the use of a retrospective self‐report questionnaire. The use of such tools could be problematic due to the possibility of recall bias. However, previous research has typically shown that individuals are more likely to under‐report the occurrence and severity of maltreatment (Shaffer et al., 2008; Williams, 1994). Furthermore, while we aimed to quantify maltreatment occurring or beginning prior to T1 (so as to examine its effect prospectively), we cannot be sure that participants were able to accurately remember the exact age (or age range) when maltreatment occurred. Moreover, many items were endorsed as lifetime, indicating that maltreatment was ongoing post T1. Fourth, the variance in some forms of maltreatment were low, and different forms of maltreatment were highly correlated, preventing assessment of the specific effects of individual forms of maltreatment. Fifth, we did not investigate the role of specific types of psychopathology in this study because of small numbers. Sixth, while we speculate that maltreatment and early‐onset psychopathology may have deleterious effects on hippocampal development, we cannot infer causal relationships due to the possibility of potential confounding factors such as genetic liability to psychopathology. Finally, the choice for the cut‐off for early and late psychiatric diagnosis was somewhat arbitrary, although matched the mean age of the middle scan time point. The aim of this grouping was to gain some insight into cause and effect in relation to hippocampal development. Investigating the effect of age of onset as a continuous variable is an alternative approach to investigating this issue, although this would of only be possible for those with a diagnosis. Thus, our approach allowed us to investigate the impact of diagnosis (present versus absent), in addition to providing some insight into the direction of associations.

In conclusion, this study investigated the effects of childhood maltreatment on the development of hippocampal subregions from early to late adolescence using a longitudinal design. We found that childhood maltreatment, and early and late onset psychiatric disorders were associated with altered development of hippocampal subregions. Further research is necessary to investigate whether these effects on hippocampal subregion development have implications for understanding risk for psychopathology and other outcomes later in life.

## Conflict of Interest

No author has any conflicts of interest to declare.
