# Supplementary material for: Childhood maltreatment, psychopathology, and the development of hippocampal subregions during adolescence
Source: Brain Behav. 2016 Nov 30;7(2):e00607. doi: 10.1002/brb3.607 (PMC5318361; doi:10.1002/brb3.607)
Supplement: Supplementary file 1 [file BRB3-7-e00607-s001.docx]

*Normative hippocampal sub-region development*

For both left (Estimate = -2.557 t= -2.637 p= 0.009) and right total hippocampal volume (Estimate = -4.456 t= -4.468 p< 0.001), there was a significant quadratic effect of age. Regarding the hippocampal sub-region volumes, a significant quadratic effect of age was found in the prediction of volumes of CA2-CA3, both left (Estimate = -1.0883 t=-2.730 p=0.007 and right (Estimate =-2.0602 t=-4.946 p=0.000), left CA4-DG (Estimate = -0.486 t=-2.336 p= 0.021), left presubiculum (Estimate = -0.683 t= -2.628 p= 0.009), left subiculum (Estimate =-0.869 t= -3.760 p= 0.000), and right CA1 (Estimate =-0.625 t=-3.427 p=0.001). There was a significant linear effect of age on the prediction of volume of the left CA1 (Estimate = 2.578 t=5.429 p= 0.000) and the left fimbria (Estimate = 1.958 t=6.742 p=0.000).

The development of some regions were moderated by sex. The right presubiculum showed a nonlinear age effect that interacted with sex (Estimate = 1.032 t= 2.403 p= 0.017); nonlinear development was significant in males (Estimate = -1.362 t=-4.306 p< 0.001), but not in females (Estimate = -0.350 t= -1.218 p=0.227). The right subiculum also showed a nonlinear age effect that interacted with sex. Nonlinear development was significant in both males (Estimate = 5.663 t= 7.305 p< 0.001) and females (Estimate = -1.075 t= -3.223 p= 0.002). Finally, there was a significant interaction between age and sex in the prediction of right fimbria volume; an age effect was significant for females (Estimate = -1.075, t=-3.223 p=0.002 ), but not for males (Estimate =0.0736 t=0.179 p=0.859).

*Non-significant female results for the effects of maltreatment on hippocampal volumes*

Figure S1. Non-significant age^2^ by childhood maltreatment effect for the right hippocampus in females.

Figure S2. Non-significant age^2^ by childhood maltreatment effect for the left CA4-DG in females.

*Non-significant male results for the effects of psychiatric diagnosis on hippocampal volumes*

Figure S3. Non-significant age^2^ by early psychiatric diagnosis effect for the right presubiculum in males.

Figure S4. Non-significant age by late psychiatric diagnosis effect for the right CA1 in males.

*Previous analysis*

We have previously published on associations between maltreatment, psychopathology and hippocampal development in the same sample (Whittle et al., 2013), however, in this work we did not investigate hippocampal sub-regions. We have also investigated the association between psychopathology (specifically, depressive disorder) and brain development in the sample (Whittle et al., 2014a). In both of these analyses, only brain imaging data from early and mid-adolescence was included. Further, we have published on the effects of other environmental factors on hippocampal development in the sample. Namely, we have investigated whether different observed measures of parenting behaviors predict hippocampal development (Whittle et al., 2014b; Whittle et al., 2016).

**References**

Whittle S, Dennison M, Vijayakumar N, Simmons JG, Yücel M, Lubman DI, Pantelis C, Allen NB. 2013. Childhood maltreatment and psychopathology affect brain development during adolescence. Journal of the American Academy of Child & Adolescent Psychiatry 52(9):940-952.

Whittle S, Lichter R, Dennison M, Vijayakumar N, Schwartz O, Byrne ML, Simmons JG,
Yücel M, Pantelis C, McGorry P. 2014a. Structural brain development and depression
onset during adolescence: a prospective longitudinal study. American Journal of Psychiatry.

Whittle S, Simmons JG, Dennison M, Vijayakumar N, Schwartz O, Yap MB, Sheeber L, Allen NB. Positive parenting predicts the development of adolescent brain structure: a longitudinal study. Dev Cogn Neurosci. 2014b Apr;8:7-17.

Whittle S, Vijayakumar N, Dennison M, Schwartz O, Simmons JG, Sheeber L, Allen NB. Observed Measures of Negative Parenting Predict Brain Development during Adolescence.
PLoS One. 2016 Jan 29;11(1):e0147774. doi: 10.1371/journal.pone.0147774.
